# Supplementary material for: Intermolecular interactions probed by rotational dynamics in gas-phase clusters
Source: Nat Commun. 2024 May 22;15:4360. doi: 10.1038/s41467-024-48822-z (PMC11111446; doi:10.1038/s41467-024-48822-z)
Supplement: Supplementary file 1 — Supplementary Information [file 41467_2024_48822_MOESM1_ESM.pdf]

Contents

Supplementary Note: Populations of *j* and *L*-states, and radical distributions along the stretch coordinate

Supplementary Note: Populations of *j* and *L*-states, and radical distributions along the stretch coordinate

As eigenstates of N_2-Ar are composed of different j and L-states, a qualitative description can be obtained by using approximate quantum numbers (n, j, L) according to the nodes along the stretch coordinate, and the dominant components of j and L-states. To obtain a direct knowledge of the relative contributions of the various modes, populations of different j and L-states of the eigenstates are provided. In Supplementary Figs. 1a and b, the populations of j and L-states corresponding to the eigenstates in Fig. 5c in the manuscript are presented. It can be seen that as the increasing of energy, the quantum number j becomes nearly rigorous and the N_2 rotation behaves like a free rotor, while L still shows a wide distribution attributing to the small rotational energy of vdW axis. Meanwhile, radical distributions of three states that contribute to the intermolecular stretching are presented in the Supplementary Fig. 1c.

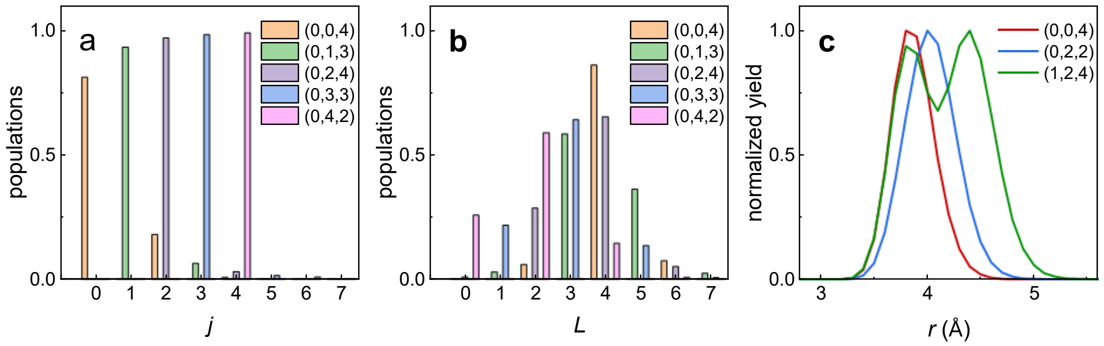

Supplementary Fig. 1 a Relative populations of j-states of eigenstates. **b** Same as **a** but for the L-states. **c** Radical distributions along the stretch coordinate r of eigenstates. The eigenstates (Fig. 5c in the manuscript) are assigned by approximate quantum numbers (n, j, L) according to the number of nodes in the stretch coordinate r, and the most populated components of j and L.
